# Supplementary figures and images for: A Computational Model of Inhibition of HIV-1 by Interferon-Alpha
Source: PLoS One. 2016 Mar 24;11(3):e0152316. doi: 10.1371/journal.pone.0152316 (PMC4807028; doi:10.1371/journal.pone.0152316)

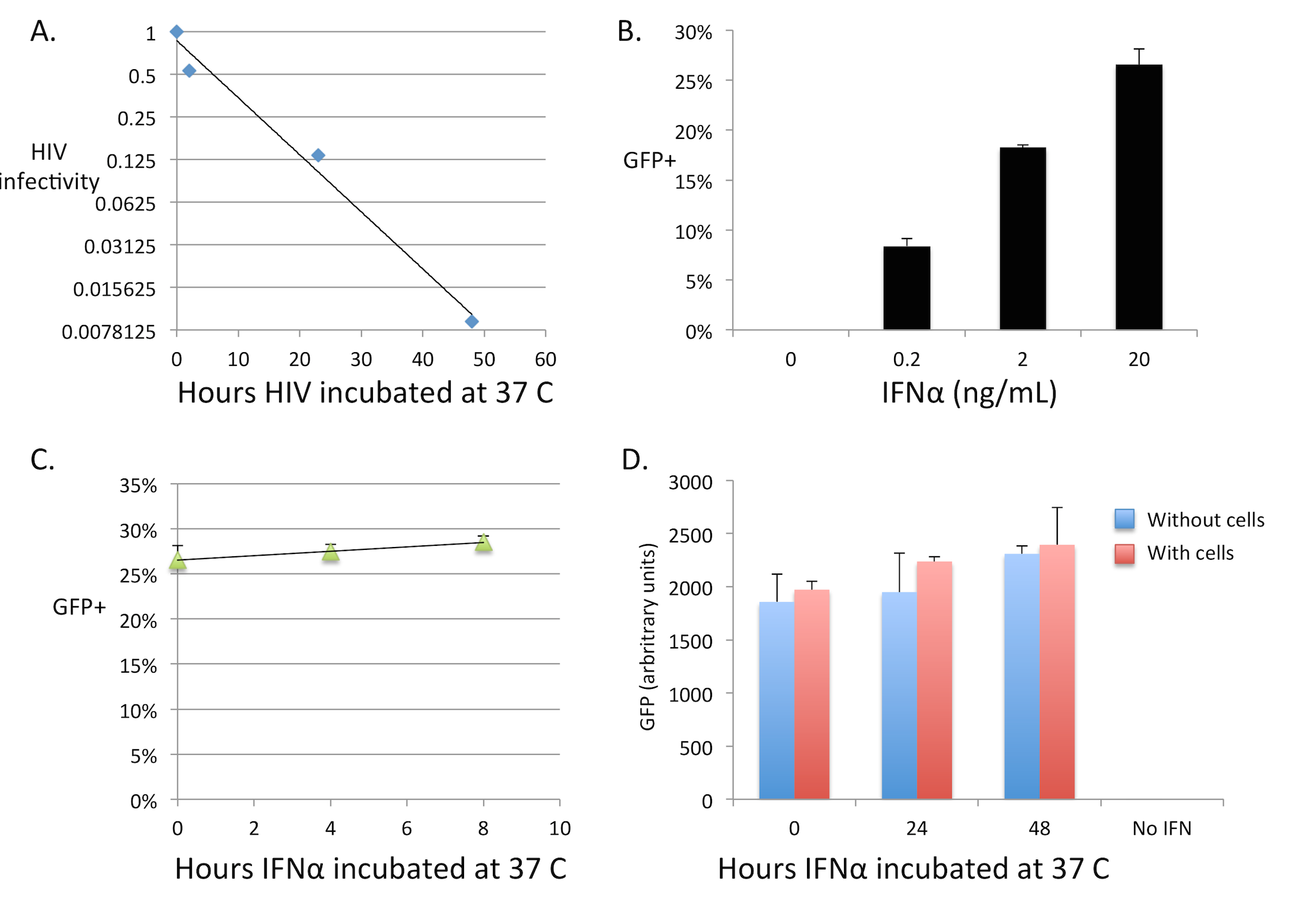

Supplement: S1 Fig — (TIF) [file pone.0152316.s001.tif]

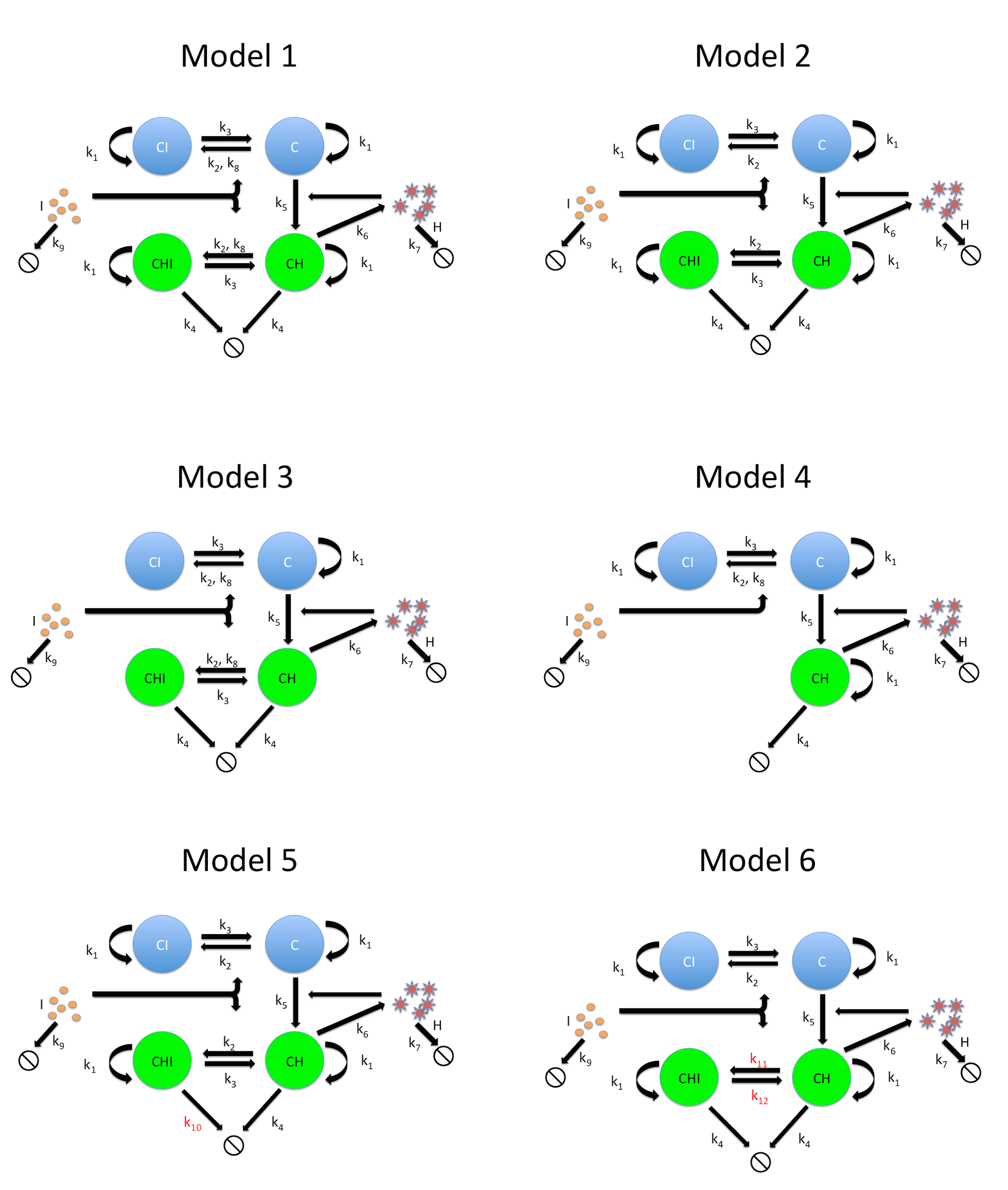

Supplement: S2 Fig — (TIF) [file pone.0152316.s002.tif]

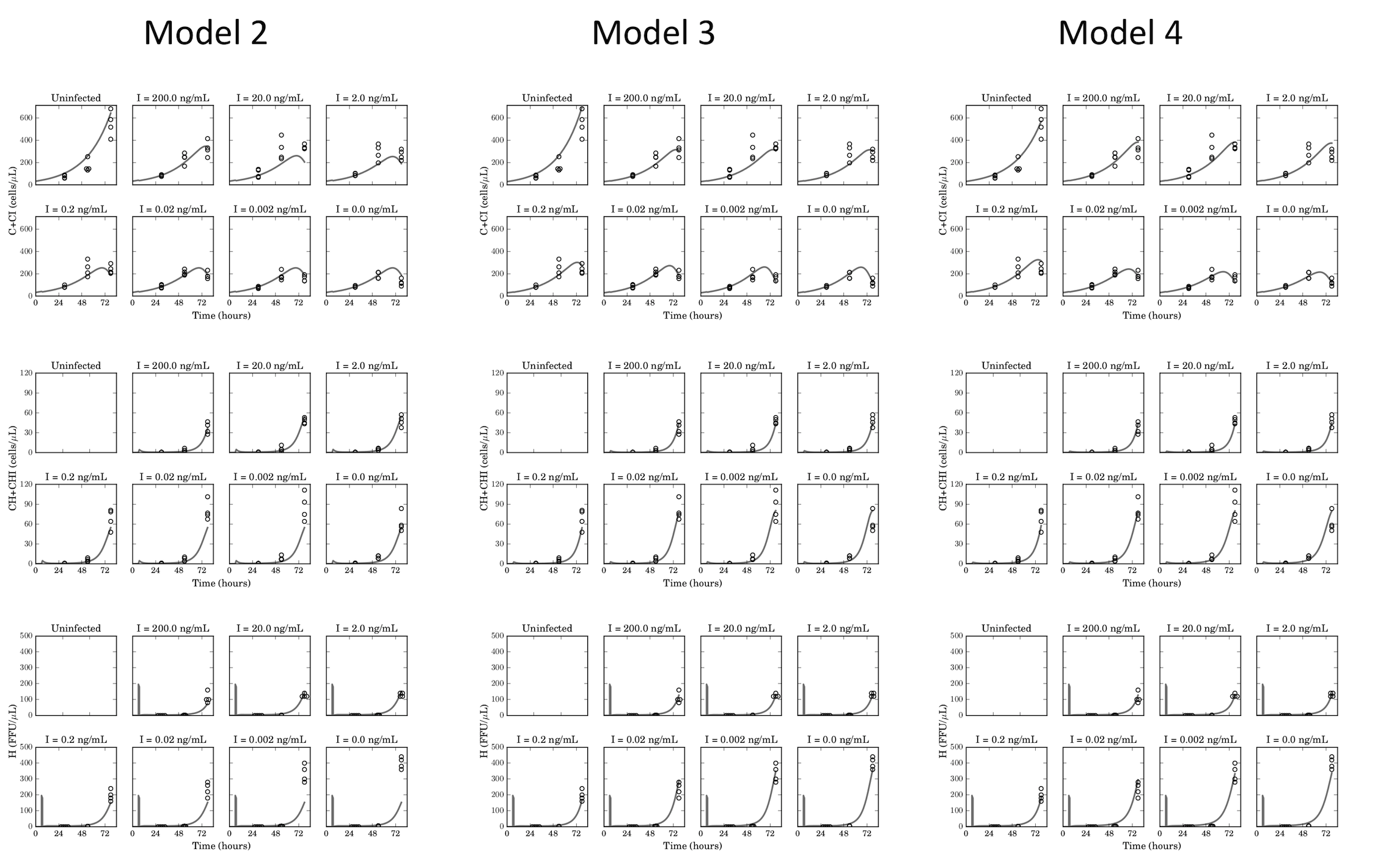

Supplement: S3 Fig — (TIF) [file pone.0152316.s003.tif]

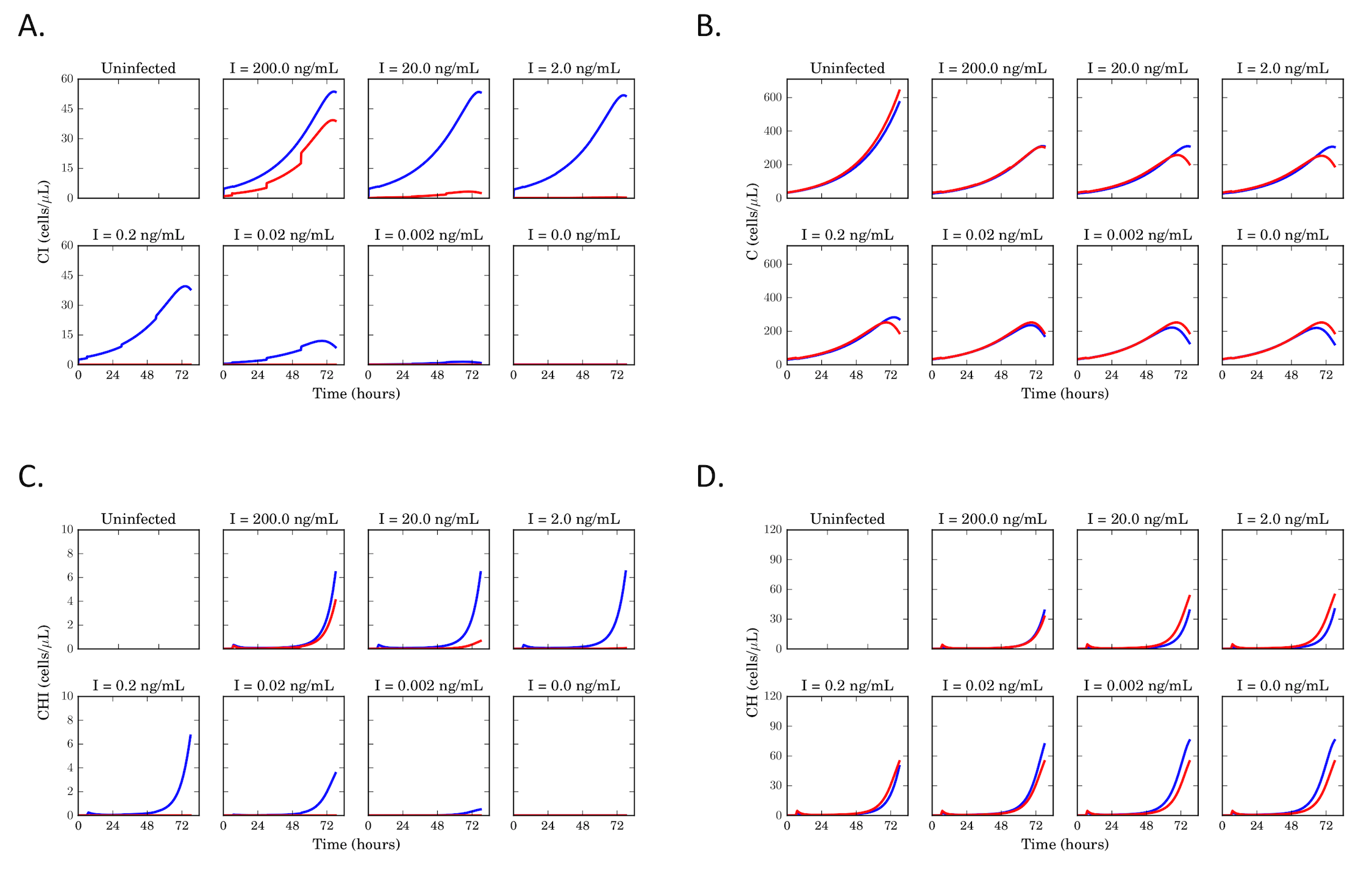

Supplement: S4 Fig — (TIF) [file pone.0152316.s004.tif]
