# Supplementary material for: A Computational Model of Inhibition of HIV-1 by Interferon-Alpha
Source: PLoS One. 2016 Mar 24;11(3):e0152316. doi: 10.1371/journal.pone.0152316 (PMC4807028; doi:10.1371/journal.pone.0152316)
Supplement: S1 File — (DOCX) [file pone.0152316.s005.docx]

**Supplemental information:**

**S1 Fig. Calculation of decay rates for HIV-1 and IFNα.**

(A) To determine the decay rate for HIV-1, a sample of HIV-1 was diluted into tissue culture media and incubated at 37C. At indicated times, samples were removed and the concentration of infectious HIV-1 was measured by focus-forming assay. Y-axis values represent the infectivity of the experimental sample as a fraction of the original infectivity of the sample. (B) An assay to determine the biological activity of IFNα samples was established by adding different concentrations of IFNα to HEK293FT cells that had been transfected with an ISRE-GFP reporter plasmid. Flow cytometry of the HEK293FT cells at 24h post exposure was then performed. Y-axis values represent the percentage of cells that were GFP+, indicating exposure to bioactive IFNα. (C) To determine the decay rate for IFNα, a sample of IFNα was diluted into tissue culture media at 20ng/mL and incubated at 37C. At indicated times, samples were removed for analysis by adding aliquots to cultured HEK293FT cells that had been transfected with an ISRE-GFP reporter plasmid. At 24h post-exposure, the cells were analyzed by flow cytometry for the percentage of cells with GFP expression. (D) To analyze the decay of IFNα activity over a longer time scale, and to examine the impact of actively dividing CD4 T cells on the decay rate for IFNα, IFNα (20ng/mL) was added to tissue culture media with or without CD4 T cells, at 37C. At times indicated, supernatant samples were removed and added to HEK293FT cells that had been transfected with an ISRE-GFP reporter plasmid. At 24h post-exposure, the GFP signal from the 293FT cells was measured using a BioTek plate reader. The Y axis represents arbitrary units of fluorescence that are proportional to the mean fluorescent intensity of the cells in each well. From these data the decay constants for HIV-1 (k_7_) and IFNα (k_9_) were determined to be 2.23 and 0 day^-1^ respectively.

**S2 Fig. Alternative model configurations.**

In addition to the basic saturating model (Model 1) and linear model (Model 2), we examined the performance of additional models in which only uninfected susceptible cells divide (Model 3), in which IFNα only impacts uninfected cells (Model 4), in which IFNα-exposed infected cells have a separate death rate from infected cells (Model 5), and in which the conversion rates to IFNα-exposed state and reversion are separate for infected and uninfected cells (Model 6).

**Table A. Parameter values for best fit from alternative model configurations.**

| **Parameter** | **Units** | **Model 1 value (95% CI)** | **Model 2 value (95% CI)** | **Model 3 value (95% CI)** | **Model 4 value (95% CI)** | **Model 5 value (95% CI)** | **Model 6 value (95% CI)** |
| --- | --- | --- | --- | --- | --- | --- | --- |
| k1 | day^-1^ | 0.886 (0.820-0.930) | 0.910 (0.842-0.956) | 0.973 (0.900-1.021) | 0.890 (0.824-0.937) | 0.921 (0.851-0.967) | 0.911 (0.865-0.956) |
| k2 | ng^-1^ day^-1^ | 45.2 (39.8-52.3) | 0.0763 (0.0544-0.1314) | 19.8 (16.5-22.9) | 9.27 (0.49-120.0) | 0.000243 (0.00009-0.0065) | 0.000001 (0-0.608) |
| k3 | day^-1^ | 261 (9.2-338) | 476 (0-29980) | 247 (8.7-1724) | 23.5 (1.2-297.2) | 0.421 (0.0-295.6) | 34.9 (0-inf) |
| k4 | day^-1^ | 5.92 (4.65-7.29) | 8.75 (6.87-10.78) | 6.60 (5.18-8.51) | 5.42 (4.26-7.00) | 8.92 (7.00-11.51) | 8.79 (6.90-10.82) |
| k5 | uL day^-1^ FFU^-1^ | 0.00991 (0.00850-0.0115) | 0.0177 (0.0147-0.0208) | 0.0121 (0.0101-0.0142) | 0.0102 (0.0088-0.0118) | 0.0183 (0.0148-0.0226) | 0.0177 (0.0147-0.0218) |
| k6 | FFU cell^-1^ day^-1^ | 19.0 (18.1-20.0) | 12.9 (11.7-13.6) | 18.2 (16.4-20.1) | 17.3 (16.0-19.1) | 12.6 (11.4-14.0) | 12.9 (11.7-14.3) |
| k7 | day^-1^ | 2.23 | 2.23 | 2.23 | 2.23 | 2.23 | 2.23 |
| k8 | ng mL^-1^ | 0.191 (0.082-0.411) | NA | 0.142 (0.061-0.363) | 0.249 (0.107-0.532) | NA | NA |
| k9 | day^-1^ | 0 | 0 | 0 | 0 | 0 | 0 |
| k10 | day^-1^ | NA | NA | NA | NA | 0.0 (0.0-inf) | NA |
| k11 | ng^-1^ day^-1^ | NA | NA | NA | NA | NA | 0.151 (0-0.214) |
| k12 | day^-1^ | NA | NA | NA | NA | NA | 426 (0-inf) |
| C_0_ | cells ul^-1^ | 32.1 (25.2-37.2) | 33.3 (29.0-39.1) | 29.4 (22.4-36.2) | 31.4 (24.6-38.7) | 33.1 (26.7-38.8) | 33.4 (26.2-38.7) |

A weighted nonlinear least squares method was used to fit experimental data to six different model configurations shown in Fig S2. For each model, parameter estimates and a 95% confidence intervals (95% CI) were determined.

**S3 Fig. Best fits for different model configurations.**

Experimental data for the concentration of uninfected (GFP-) cells (A), infected (GFP+) cells (B), and infectious HIV-1 (C) was fitted with Models 2, 3 and 4. Open circles represent independent experimental data points, while for each species, the solid black lines represent the model’s best fit.

**S4 Fig. Estimated concentrations for individual cell species during timecourse infection.**

The estimated concentrations for individual cell species CI (A), C (B), CHI (C) and CH (D) over the timecourse of infection were calculated for each timepoint from the best fits for Model 1 (blue lines) and Model 2 (red lines).

**Table B. Fit error with non-zero IFNα decay value.**

|  | **Model 1** | **Model 2** |
| --- | --- | --- |
| Fit error | 278.3 | 1368.0 |

To determine whether our assumption of zero decay rate for IFNα influenced the conclusions of our study, we performed fitting with the half-life for IFNα arbitrarily set to one day and compared the fit errors between Model 1 and Model 2. We determined that Model 1 provided a significantly more accurate fit than Model 2, similar to our fit with zero decay for IFNα. This demonstrates that the superior performance of Model 1 is not dependent on the assumption of a negligible decay rate for IFNα.

**Table C. Fit error with virus consumption by cells.**

|  | **No virus consumption** | **With virus consumption** |
| --- | --- | --- |
| Fit error | 276.6 | 290.22 |

To determine whether virus consumption could significantly affect the outcome of model fitting, the fir error of Model 1 was compared to a variant that included cell-dependent consumption of HIV virions (by adding the term -k5*(C+CH)*H to the dH/dT equation). The fit error in this case was found to be similar to the fit error for Model 1 and other saturating model variants.
